# Proceedings of the 2^nd^ Symposium “Autoimmune Diseases -Clinical Unmet Needs in Systemic Autoimmune Diseases Guide Clinical, Translational, and Basic Research”

**DOI:** 10.31138/mjr.190126.una

**Published:** 2026-03-01

**Authors:** Loukas Chatzis, Ioanna Myrto Theofani, Ioanna Evdokia Galani, Panagiota Palla, Alexandra Koutsogianni, Dimitrios Palamidas, Panagiotis Panagopoulos, Evangelia Dafni Prifti, Evangelos Andreakos, Athanasios G. Tzioufas

**Affiliations:** 1 Pathophysiology Department, Athens School of Medicine, National and Kapodistrian University of Athens, Athens, Greece;; 2 Research Institute for Systemic Autoimmune Diseases, Athens, Greece;; 3 Laboratory of Immunobiology, Centre for Clinical, Experimental Surgery and Translational Research, Biomedical Research Foundation of the Academy of Athens, Athens, Greece

**Keywords:** symposium, autoimmune diseases, Sjogren diseases, systemic lupus erythematosus, vasculitis, proceedings

## INTRODUCTION

Although bench and bedside oriented publications are being generated at an unprecedented pace, only a relatively small proportion translate into meaningful clinical impact. The low-dimensional technologies traditionally mastered by physicians working in close collaboration with scientists that have been instrumental in shaping modern medical practice are increasingly being replaced by high-dimensional single-cell and spatial technologies. These approaches integrate enormous multimodal datasets in an effort to bridge the gap between tissue- or cell-level endotypes and specific clinical phenotypes. However, the widespread adoption of such technologies has, paradoxically, widened the gap between clinicians and researchers, effectively placing the concept of the clinician–scientist into a state of hibernation.

As a call to reawaken this paradigm, we initiated the symposium *“Autoimmune Diseases: Clinical Unmet Needs in Systemic Autoimmune Diseases Guide Clinical, Translational, and Basic Research,”.* The success of the first meeting^1^ paved the way for a second symposium, held on October 10–11, 2025, and organised by the Research Institute for Systemic Autoimmune Diseases with the support of the Medical School of Athens and the Biochemical Research Foundation of the Academy of Athens, aimed to bring together renowned Greek and international clinicians and researchers. This event, chaired by Professor Athanasios G. Tzioufas and Dr. Evangelos Andreakos, was held under the auspices of the Medical School of the National and Kapodistrian University of Athens. The meeting fostered a dynamic exchange of knowledge focused on a shared goal: improving patient outcomes through a deeper understanding of disease mechanisms. The symposium drew more than 150 participants, including physicians, biologists, and researchers at various academic levels, from professors to students. A summary of the presentations is provided below, grouped by scientific field.

## SYSTEMIC VASCULITIDES

In his plenary lecture, Professor Patrice Caccoub addressed cryoglobulinaemic vasculitis (CV), a small-vessel vasculitis characterised by systemic involvement affecting multiple organs, most commonly the skin, joints, peripheral nervous system, and kidneys, while less frequently involving the central nervous system and the heart. From a pathogenetic perspective, cryoglobulinemia is classified according to the type of circulating cryoglobulins. Type I cryoglobulinemia, typically associated with underlying B-cell lymphoproliferative disorders, does not represent a true immune-mediated vasculitis. Its clinical manifestations result primarily from vascular occlusion caused by circulating monoclonal immunoglobulins, leading to acral ischemia and necrosis, painful oedema, hyperviscosity syndrome, and severe Raynaud’s phenomenon. In contrast, type II and type III cryoglobulinaemias, collectively referred to as mixed cryoglobulinaemias, are characterised by immune complex–mediated small-vessel vasculitis, characterised histologically by perivascular inflammatory infiltrates and complement deposition.^2^ The most frequent clinical features include palpable purpura, arthralgias, peripheral neuropathy, and renal involvement, the latter representing the main determinant of prognosis. Mixed cryoglobulinemia is associated with a broad spectrum of underlying conditions, most notably chronic hepatitis C virus infection, autoimmune diseases such as Sjögren’s syndrome (SjD) and systemic lupus erythematosus (SLE), and B-cell lymphoproliferative disorders.^3^ Hepatitis C virus infection was presented as a paradigmatic model to illustrate the underlying pathogenetic mechanisms.^4^ Therapeutic strategies are guided by the underlying aetiology, with antiviral therapy for HCV-related disease, immunosuppression combined with B-cell–depleting agents such as Rituximab for non-infectious mixed cryoglobulinemia, and targeted treatment of the haematologic disorder in type I disease, while plasma exchange is reserved for severe or life-threatening manifestations.^5^

Professor Little addressed the issue of relapse prediction in anti-neutrophil cytoplasmic antibodies (ANCA)-associated vasculitis (AAV). Relapse remains a major challenge in the long-term management of these diseases, particularly when deciding on the duration and intensity of maintenance therapy. Traditional baseline predictors of relapse, such as ANCA specificity, organ involvement, and induction regimen, provide only a rough estimate of risk and show considerable heterogeneity across treated populations. While PR3-ANCA positivity, preserved renal function, and non-dialysis status are consistently associated with higher relapse risk,^6,7^ outcomes in the modern rituximab era are more nuanced. Increasing evidence highlights the importance of dynamic, post-induction biomarkers to refine relapse prediction. These include the evolution of haematuria, achievement and persistence of ANCA negativity, patterns of B-cell repopulation, and markers of ongoing myeloid activation such as calprotectin and activated monocytes.^7^ Immunological signatures reflecting T-cell exhaustion appear protective, whereas persistent immune activation portends relapse.^8^

Emerging data suggest that early plasmablast enrichment after B-cell depletion and urinary immune cell profiles may signal subclinical renal inflammation before overt relapse.^9,10^ Importantly, relapse risk estimation directly influences clinical decisions regarding rituximab re-dosing and treatment withdrawal. Novel approaches using machine learning models that incorporate longitudinal data and memory of prior disease events show promise in improving individualised risk stratification. Collectively, Prof. Little concluded that integrating static and dynamic biomarkers into time-sensitive decision frameworks may enable more personalised, safer maintenance strategies in AAV.

### Systemic Lupus Erythematosus

Professor Liossis proceeded to discuss new and emerging therapies for SLE. B cells are major orchestrators of SLE pathogenesis due to hyperactivity, autoantibody production, antigen presentation, and cytokine secretion. This provides the biological rationale for B-cell–directed therapies as a cornerstone of emerging lupus treatments. Obinotuzumab is a type II anti-CD20 monoclonal antibody with more potent B-cell depletion compared to Rituximab and may offer improved efficacy in selected patient subgroups.^11^ Professor Liossis focused on the growing enthusiasm for advanced cellular therapies, particularly anti-CD19 CAR T-cell therapies. More than 50 protocols have been conducted worldwide and over 100 patients with connective tissue diseases have been treated.^12^ However, major unanswered questions remain regarding optimal CAR T-cell dosing, cellular composition, long-term efficacy, and whether clinical benefit is driven by CAR T-cells themselves or by the mandatory myeloablative conditioning regimens. Safety concerns, including the risk of secondary malignancies and the poorly defined concept of “deep immune system reset,” remain substantial.^13^ Alternative strategies such as bispecific T-cell engagers (BiTEs), including CD19-CD3 constructs, offer promising efficacy with potentially lower toxicity. Additionally, engineered regulatory T-cell (Treg) therapies targeting lupus-specific antigens represent a novel and potentially safer approach, showing encouraging effects on autoantibody reduction, tissue inflammation, and lupus nephritis.^14^ Overall, while next-generation immunotherapies hold significant promise in SLE, challenges related to disease heterogeneity, outcome assessment, and long-term safety must be carefully addressed.

Professor Bertsias, in his lecture, focused on the pathogenetic mechanisms of SLE.^15^ SLE is a complex autoimmune disease driven by the interplay of genetic, epigenetic, environmental, and stochastic factors, involving multiple immune cell types and molecular pathways. Recent advances have highlighted the pivotal role of innate immunity, particularly neutrophils and plasmacytoid dendritic cells, in both disease initiation and perpetuation. Low-density granulocytes are expanded in SLE and promote autoimmunity and tissue injury through enhanced NETosis and the release of mitochondrial DNA, leading to sustained type I interferon (IFN) activation.^16,17^ Accumulating genetic evidence identifies more than 120 risk loci, reinforcing the concept of SLE as an interferon-driven disease characterised by increased nucleic acid sensing. Novel genetic and functional studies implicate key regulators such as TLR7, UNC93B1, PLD4, and IRF5, providing direct links between pathogenic mechanisms and emerging therapeutic targets.^18^ Interferons display differential bioactivity, with type I IFN associated with baseline disease and type II/III IFNs contributing to active or severe disease states. Advanced single-cell and transcriptomic analyses reveal cell-type–specific contributions to disease activity, metabolic reprogramming of immune cells, and organ-specific pathogenic signatures.^19^ Adaptive immunity is equally critical, with specialised T follicular helper and peripheral helper T cells driving highly pathogenic B-cell responses and autoantibody production.^20^ Importantly, non-immune parenchymal cells, such as keratinocytes, actively contribute to inflammation through local interferon production. Professor Bertsias concluded that these insights refine current concepts of SLE pathogenesis and provide a foundation for biomarker discovery and targeted therapeutic strategies.

### Systemic Sclerosis

In his presentation, Professor Theodoros Dimitroulas discussed management strategies and future perspectives in systemic sclerosis (SSc), highlighting the heterogeneity that characterises this complex autoimmune disease.^21^ He emphasised that variability in clinical manifestations and disease progression demand individualised treatment approaches rather than standardised therapeutic algorithms.

Prof. Dimitroulas highlighted inflammation as a key factor in treatment selection and referred to studies examining the genes involved in SSc, which identified different subtypes, such as inflammatory, fibroproliferative, limited and normal-like.^22^ Individuals with the inflammatory subtype appeared to respond better to immunosuppressive treatments like mycophenolate mofetil.^23^

These findings underscore the importance of stratifying patients by their molecular subtype for both treatment and research purposes. Focusing on interstitial lung disease (ILD), a major cause of morbidity and mortality in people with SSc, he reviewed evidence that immunosuppressive drugs, biologics such as rituximab and tocilizumab, and antifibrotic treatments with nintedanib can be beneficial.^24^ He strongly emphasised the need for early detection, prompt intervention, and combination therapies to preserve lung function and slow disease progression. The role of tocilizumab in early inflammatory SSc-ILD was also discussed,^25^ along with the integration of antifibrotic therapy into therapeutic strategies. Professor Dimitroulas noted that current guidelines recommend starting with combination therapy for SSc-associated PAH and mentioned emerging new drugs.^26^ Importantly, he pointed out that anticoagulants are no longer recommended for SSc-PAH, as they may increase mortality.^27^

The presentation concluded by reinforcing the need for precision medicine in SSc, integrating clinical evaluation with molecular and inflammatory profiling to optimise therapeutic decisions and improve long-term outcomes.

During his talk, Professor Dimitrios Daoussis provided an in-depth analysis of the pathogenic mechanisms underlying SSc, focusing on the dynamic interplay between vasculopathy, autoimmunity, and fibrosis. He emphasised that vascular injury represents one of the earliest and most critical events in disease initiation, often preceding overt inflammation and fibrotic remodelling.

Professor Daoussis highlighted endothelial cell apoptosis as a primary pathogenic trigger, supported by experimental evidence from animal models and human tissue studies.^28^ Early vascular damage leads to impaired angiogenesis, chronic tissue hypoxia, and recruitment of immune cells, setting the stage for sustained inflammation. Autoimmune mechanisms were discussed in depth, with particular emphasis on the presence of autoantibodies and their association with distinct clinical phenotypes.

The central role of B cells in disease pathogenesis was strongly emphasised. Evidence from murine models and clinical studies demonstrated that chronic B-cell activation, altered signalling thresholds, and autoanti-body production contribute to immune dysregulation and fibrosis.^29^ Clinical responses observed with B-cell–depleting therapies further support their pathogenic relevance.

Fibrosis, the hallmark of systemic sclerosis, was presented as a multifactorial process. In addition to resident fibroblasts, circulating fibrocytes and cells undergoing endothelial- and epithelial-to-mesenchymal transition were discussed as contributors to extracellular matrix accumulation.^30^ The speaker highlighted the heterogeneity of fibroblast populations, including disease-associated subsets with distinct profibrotic programs.

At the conclusion of his speech, Prof. Daoussis underlined the importance of early diagnosis and aggressive treatment with combination of immunosuppressants. He also suggested that immunomodulatory strategies may be most effective during inflammatory phases, whereas targeted antifibrotic approaches may be required in advanced disease.^31^

### Sjögren’s Disease

Professor Michele Bombardieri’s keynote presentation outlined the pathophysiology of ectopic lymphoid structures (ELS) and their essential function in autoimmune diseases. He explained that ELS, commonly known as tertiary lymphoid structures, look like secondary lymphoid organs in both appearance and function and arise in tissues that are chronically inflamed. These formations display networks of follicular dendritic cells, organised B- and T-cell regions, and responses similar to those in germinal centres, suggesting ongoing immunological activity initiated by antigens.^32,33^

Prof. Bombardieri stated that ELS assist local B cells in maturing and undergoing modifications that result in the production of high-affinity autoantibodies associated with disease. He emphasised the functions of T follicular helper (Tfh) and peripheral helper T (Tph) cells, which transmit crucial signals via IL-21, ICOS-ICOSL, and CD40-CD40L. He emphasised that stromal cells and chemokine gradients such as CXCL13, CCL19, and CCL21 are crucial for structuring lymphoid formations in inflamed tissues.^34,35^

The talk also addressed the importance of metabolic and epigenetic reprogramming in influencing ELS function. Altered lipid metabolism and heightened glycolysis have been shown to influence immune cell development and cytokine production, thereby affecting local immunological responses. These findings suggest that metabolic pathways might serve as novel therapeutic targets that disrupt the activity of pathogenic ELS. Importantly, Professor Bombardieri discussed the clinical relevance of ELS in diseases such as rheumatoid arthritis and Sjögren’s syndrome. A high burden of ELS has been associated with more severe disease phenotypes, continuous autoantibody production, and an increased risk of lymphomagenesis, specifically mucosa-associated lymphoid tissue lymphoma.^36,37^

Transcriptomic analysis revealed various molecular signatures associated with ELS-positive tissues, validating their role as biomarkers for classifying patients and forecasting treatment responses.^38^

In conclusion, the speaker highlighted that ELS serve as active triggers of autoimmunity rather than just epiphenomena of chronic inflammation. To develop targeted therapeutics and precision medicine approaches for autoimmune diseases, it is essential to understand the molecular mechanisms that regulate their production and maintenance.

In his lecture, Asst. Prof. Andreas Goules focused on recent advances in the understanding of SjD pathogenesis, emphasising the complex interplay between innate and adaptive immune mechanisms. He highlighted that activation of innate immunity, particularly type I and type II interferon pathways, represents an early and critical event in disease development.^39^

Dr. Goules discussed evidence demonstrating increased interferon-stimulated gene expression in peripheral blood and salivary gland tissues, supporting the concept of interferon-driven immune activation.^40^ These interferon signatures were presented as biomarkers of disease activity and predictors of clinical outcome.

The central role of B cells was emphasised, particularly their contribution to autoantibody production and glandular inflammation. Dr. Goules highlighted the formation of ectopic lymphoid structures within salivary glands, which provide a permissive microenvironment for antigen-driven B-cell maturation and affinity maturation.^41,42^ Elevated BAFF levels were discussed as a key factor promoting B-cell survival and persistence.^43^ The speaker further addressed advances in molecular stratification using transcriptomic and proteomic approaches. Distinct molecular endotypes of SjD were described, offering opportunities for precision medicine and targeted therapy.^44^

The lecture concluded with a discussion of emerging therapeutic strategies targeting B cells, interferon signalling, and co-stimulatory pathways, underscoring the potential for biomarker-guided treatment approaches.^38,45^

Sjögren’s disease pathogenesis is complex, involving multiple cell types and critical interactions between epithelial and immune cells across several molecular pathways. Multi-omics and tissue-level molecular stratification are central to precision medicine, enabling the discovery of reliable and clinically meaningful biomarkers.

In his lecture, Dr. Loukas Chatzis reviewed the therapeutic journey of SjD, highlighting the challenges and lessons learned from past clinical trials as well as emerging treatment strategies. He emphasised that disease heterogeneity and limitations of outcome measures have been major barriers to demonstrating therapeutic efficacy.^46^

Dr. Chatzis discussed early clinical trials that relied on non-validated endpoints and unselected patient populations, resulting in disappointing outcomes despite strong biological rationale. These experiences under-scored the need for improved trial design and more precise alignment between therapeutic targets and underlying disease mechanisms.

The speaker reviewed advances in the understanding of SjD pathophysiology, particularly the central role of B cells and co-stimulatory pathways. Therapeutic strategies targeting BAFF and BAFF receptor signalling were highlighted, with encouraging phase 2 data demonstrating improvements in systemic disease activity and glandular function.^47^ Inhibition of Bruton’s tyrosine kinase and targeting the CD40–CD40L axis were also discussed as promising approaches.^48–50^

A key challenge emphasised was the disconnect between objective disease activity indices, such as ESSDAI, and patient-reported outcomes, including fatigue and pain. Dr. Chatzis stressed that future trials must better capture patient experience while maintaining robust biological endpoints.^51^

The lecture concluded by highlighting disease heterogeneity, mismatches between objective measures and patient-reported outcomes, and high placebo responses complicate treatment evaluation. More precise immune pathway targeting and better biomarkers are needed to enable effective, personalised therapies.

### Idiopathic Inflammatory Myopathy

Professor Mavragani gave a lecture on Idiopathic inflammatory myopathies (IIMs) which represent a heterogeneous group of rare autoimmune disorders primarily affecting skeletal muscle, but with frequent involvement of other organs such as the skin, lungs, joints, gastrointestinal tract, and myocardium.^52^ Recent advances have substantially refined our understanding of their pathogenesis, highlighting the interplay between genetic susceptibility, environmental factors, and immune dysregulation. Myositis-specific and myositis-associated autoantibodies not only aid in diagnosis and patient stratification but also appear to play a direct pathogenic role by targeting nuclear or cytoplasmic autoantigens and altering key molecular pathways within muscle fibres. Distinct genetic associations, particularly involving HLA-DRB1 alleles and variations of complement genes, further support disease heterogeneity across myositis subtypes.^53^ Environmental factors, including viral exposures and medications, UV light and toxins, may influence autoantibody expression and disease onset.^54^ As Prof. Mavragani highlighted, based on histopathological and transcriptomic studies, there are distinct immune signatures among different forms of myositis, ranging from interferon-driven perifascicular injury in dermatomyositis to cytotoxic T-cell–mediated muscle damage in polymyositis and inclusion body myositis.^55^ Type I interferon pathways, particularly interferon-β, emerge as central drivers in specific subgroups, including dermatomyositis and anti-MDA5–associated interstitial lung disease.^56^ These mechanistic insights have guided novel therapeutic strategies targeting autoantibodies, B cells, T cells, and complement pathways, underscoring a shift toward more personalised and mechanism-based treatment approaches in inflammatory myopathies.

### Spondyloarthritis

Assistant Professor George Fragoulis presented the latest advances in the treatment of spondyloarthritis (SpA), a family of overlapping entities including ankylosing spondylitis (AS), psoriatic arthritis (PsA), reactive arthritis (ReA) and spondyloarthritis with inflammatory bowel disease (SIBD). First, he presented the recently defined term of early axial spondyloarthritis (early axSpA) describing patients with a diagnosis of axSpA with duration of axial symptoms of less than 2 years,^57^ and he stressed on the importance of early initiation of treatment. Then, the speaker presented the available treatment modalities for SpA patients, including inhibitors of TNF, IL-17, IL-23, IL-12/IL-23, PDE4 and JAK inhibitors, and he raised the question of whether new drugs or new strategies are needed. He proceeded to discuss the role of IL-23 inhibition in PsA. He reported the results of preclinical animal model studies showing that although IL-23 drives the development of psoriasis and psoriatic arthritis in mice,^58^ it appears to participate in the initiation and not in the perpetuation of experimental SpA in rats.^59^ A recent retrospective cohort study of patients with psoriasis has shown that treatment with IL-12/23 or IL-23 inhibitors was associated with less risk of development of inflammatory arthritis compared to TNF inhibitors.^60^ On the other hand, although IL-23 drives IL-17 production by adaptive lymphocytes including Th17 cells, IL-17 is also produced by innate-like lymphocytes, independently from IL-23. A recent analysis of IL-17 inhibitor bimekizumab and IL-23 inhibitor risankizumab trials in PsA, using matching-adjusted indirect comparisons (MAIC), has found that bimekizumab showed greater likelihood of efficacy regarding arthritis outcomes.^61^ Then, Asst. Prof. Fragoulis proceeded to discuss how different SpA manifestations and comorbidities affect choice of treatment strategy. Thus, hidradenitis suppurativa is more common in SpA patients compared to the general population and TNF inhibitor adalimumab and IL-17 inhibitors secukinumab and bimekizumab are effective treatment options.^62^ Furthermore, adipose tissue is an inflammatory tissue and obesity is characterised by increased production of pro-inflammatory cytokines, such as TNF, IL-17, and by M1 macrophage polarisation.^63^ Thus, a systematic review and meta-analysis has demonstrated that obesity is a predictor of worse response to TNF inhibition in patients with immune-mediated inflammatory diseases, including SpA and PsA.^64^ Interestingly, ongoing trials study concomitant treatment with IL-17 inhibitors and glucagon-like peptide-1 (GLP-1) receptor agonists, in PsA patients who are obese or overweight.

Dr. Gkikas Katsifis talked about the pathogenetic aspects of T helper 17 cells (Th17) related diseases. First, he revisited the differentiation of the Th17 cell lineage. Following antigen presentation by antigen presenting cells and under the influence of specific cytokines, undifferentiated T helper (Th) cells differentiate into different lineages. Although TGF-β promotes the development of regulatory T (Treg) cells, the same cytokine (TGF-β) in the presence of IL-6, with the later contribution of IL-23, inhibits Treg production and stimulates the development of Th17 cell lineage. Th17 cells produce IL-17, more specifically IL-17A and IL-17F, which play an important role both in protective immunity and in immunopathology. IL-17 activates neutrophils and macrophages, while IL-17 together with IL-22 enhances epithelial barrier function, by stimulating the expression of tight junction proteins and antimicrobial peptides.^65^ Interestingly, Th17 and Treg cells present plasticity, being able to interconvert and acquire mixed features, depending on their microenvironment. Plasticity between Th17 and Treg compartments is mediated by transcription factors and epigenetic modifications, such as chromatin reorganisation.^66^ Dr. Katsifis proceeded to discuss the role of IL-17 and Th17 lineage in different immune mediated diseases. In SpA, different innate and adaptive immune cells are located in the enthesis, including innate lymphoid cells, gamma delta T cells, natural killer cells and Th cells. These cells overproduce IL-17 and IL-22 in patients with SpA, either following stimulation by IL-23 or independently of IL-23. IL-17 promotes entheseal inflammation while IL-22 promotes osteoproliferation.^67^ In multiple sclerosis (MS) and in neuromyelitis optica (NMO), Th1 and Th17 cells infiltrate the central nervous system, alongside B cells, at areas with increased blood-brain barrier permeability, wherein they produce pro-inflammatory cytokines, leading to further inflammatory cell infiltration and tissue damage.^68^ Furthermore, IL-17 producing T cells have been identified in minor salivary gland biopsies of SjD patients and they are disproportionately increased as compared to Treg cells, leading to increased levels of IL-17 in the plasma.^69^

### Co-morbidities in Rheumatology

Professor Maria Tektonidou’s lecture focused on atherosclerosis in systemic lupus erythematosus (SLE) and in antiphospholipid syndrome (APS). First, she discussed about accelerated atherosclerosis and increased risk for cardiovascular disease (CVD) in SLE patients, leading to increased mortality. Recent studies have demonstrated a twofold to threefold higher risk for myocardial infarction and stroke in SLE patients and two times higher cardiovascular mortality compared to general population.^70^ CVD risk in SLE is higher in young patients, during the first years after disease onset and it is associated with lupus nephritis, high disease activity, positive antiphospholipid antibodies and treatment with glucocorticoids. A chronic inflammatory state in SLE is linked to accelerated atherosclerosis, characterised by immune cell activation and inflammation driven plaque formation. Furthermore, traditional cardiovascular risk factors are contributing to increased CVD risk in SLE. A number of imaging tools are increasingly used for cardiovascular risk assessment in SLE patients, including mainly carotid and femoral ultrasound and also pulse wave velocity and computed tomography (CT) coronary angiography. In a recent cross-sectional study, detection of atherosclerotic plaques by carotid and femoral ultrasound in SLE patients reclassified 20–30% of patients from non-high to high CVD risk, demonstrating that clinical risk scores underestimate CVD risk in these patients.^71^ Then, Professor Tektonidou moved on to discuss cardiovascular risk in APS and commented on the interplay among various antiphospholipid antibody mediated pathways, leading to thromboinflammation and accelerated atherosclerosis.^72^ Furthermore, traditional risk factors are more prevalent in APS patients compared to patients with RA and are associated with major CVD events.^73^ Similar to SLE patients, clinical CVD risk scores underestimate CV risk in APS patients and detection of atherosclerotic plaques by vascular ultrasound results in risk reclassification in 40–50% of APS patients.^74^ Next, Prof. Tektonidou stressed on the importance of cardiovascular risk management in SLE and APS, by means of disease activity control, use of hydroxychloroquine, reduction of glucocorticoid dose and maintenance of blood pressure lower than 130/80 mmHg in SLE patients, as well as lipid treatment in SLE and APS patients. Furthermore, she commented on low attainment of CVD risk targets in hypertension and lipid treatment in SLE and APS patients according to recent studies.^75^

Dr. Gkikas Katsifis talked about the pathogenesis and treatment of psoriatic arthritis (PsA). First, he discussed about the interplay between genetic, environmental, and immune factors in PsA. Genetic susceptibility in PsA is derived both from HLA genes, such as HLA-Cw6 and HLA-B27, and from genes outside the major histocompatibility (MHC) region, such as interleukin-12B (IL-12B) and IL-23 receptor (IL-23R).^76^ On the other hand, infections, injuries and obesity are environmental factors which have been associated with PsA development.^77^ Furthermore, dysregulated immune responses, involving immune cells and a number of cytokines, contribute to the pathogenesis of PsA, including the IL-23/ IL-17 and TNF pathways. Innate and adaptive immune cells located in the enthesis produce IL-17 and IL-22, either after stimulation by IL-23 or independently of IL-23. IL-17 acts on synovial fibroblasts and osteoclast progenitors, leading to synovial inflammation and development of bone erosions, while IL-22 acts on osteoblast progenitors leading to abnormal bone formation.^78^ Interestingly, pro-inflammatory cytokines, including TNF, IL-17, IL-23, may drive T regulatory cell plasticity toward a T helper 17 like phenotype, characterised by IL-17 and TNF production.^79^ Then, Dr. Katsifis went on to discuss treatment of PsA. PsA often presents with multiple manifestations, including peripheral arthritis, axial involvement, dactylitis, enthesitis, skin psoriasis and nail psoriasis. There is a wide armamentarium of treatment modalities available, targeting TNF, IL-17, IL-23, IL-12/-23 receptor, Janus kinases and phosphodiesterase-4. Treatment selection for each patient needs to take into account PsA manifestations. TNF, IL-17, IL-23 and JAK inhibitors are effective for peripheral arthritis and these agents appear to have similar efficacy on joint involvement. IL-17 and IL-23 inhibitors are more effective for skin psoriasis compared to TNF and IL-12/23 inhibitors,^80^ while risankizumab (IL-23 inhibitor) has been found more effective than secukinumab (IL-17 inhibitor) for skin involvement.^81^ IL-17 and IL-23 inhibitors are effective for nail psoriasis,^82^ while TNF, IL-17 and JAK inhibitors are effective for axial disease. Furthermore, comorbidities are frequent in patients with PsA and need to be taken into account at treatment selection.^83^

Assistant Professor Christos Koutsianas gave a lecture about comorbidities in anti-neutrophil cytoplasmic antibodies (ANCA) associated vasculitis (AAV). First, he discussed about increased mortality of AAV patients compared to general population, commenting that mortality is increased in myeloperoxidase (MPO) ANCA positive patients compared to proteinase-3 (PR3) ANCA positive patients.^84^ Notably, survival of AAV patients has improved during the last decades, with decrease of vasculitis-, cardiovascular- and infection-associated deaths but stable malignancy-associated deaths.^85^ However, patients with AAV accumulate damage over time in multiple categories (renal, otolaryngological and treatment related),^86^ exhibiting a shift over the years to survivors with chronic problems and damage. Next, Asst. Prof. Koutsianas proceeded to discuss different comorbidities in AAV. Patients with AAV present increased risk of myocardial infarction and ischemic heart disease, especially patients with microscopic polyangiitis (MPA), positive MPO-ANCA, high disease activity, greater age at disease diagnosis and patients in the three months following diagnosis.^87^ AAV appears to be associated with a hypercoagulable state, characterised by hyperactivation of the intrinsic coagulation pathway, increased activation of the complement cascade and increased neutrophil extracellular trap (NET) formation.^88^ AAV patients present also increased risk for venous thromboembolism^89^ and ischemic stroke.90 Furthermore, patients with AAV present increased risk for serious infections, particularly during the induction period at the first six months after diagnosis,^91^ and in patients with lung and kidney involvement, end stage renal disease, leukopenia and treatment with intravenous pulse corticosteroids, cyclophosphamide, rituximab and plasmapheresis.^92^ Furthermore, malignancy risk is increased in AAV patients, for all types of cancer. Increased malignancy risk is associated with cyclophosphamide treatment, whereas this is not the case for rituximab.^93^ Notably, patients with systemic vasculitis present increased risk for osteoporosis and for fragility fractures (94). Asst. Prof. Koutsianas concluded that improvements in the treatment of AAV have led to a paradigm shift, where patients live longer, but accumulate more damage and comorbidities over time, necessitating awareness, identification and management of these sequelae.

## BASIC AND TRANSLATIONAL IMMUNOLOGY

### Systemic autoinflammatory diseases

Dr. Soumelis presented the ImmunAID project, a large European collaborative effort aimed at improving the diagnosis and management of systemic autoinflammatory diseases (SAID) through integrative multi-omics approaches. The project recruited patients from a wide clinical spectrum, from monogenic to genetically undiagnosed forms of SAID, alongside healthy and family members controls, in total comprising over 600 participants. A variety of data modalities, including genomics, transcriptomics, proteomics, lipidomics, microbiomics, cytokine and chemokine profiling, as well as deep immunophenotyping, were newly generated from the participants’ peripheral blood mononuclear cells (PBMC) to enable systems-level understanding of disease mechanisms and to identify novel biomarkers and classification frameworks. Dr. Soumelis in his presentation showcased the example of two diseases for which this type of analysis was applied.

The first case study focused on Still’s disease, encompassing both Adult Onset Still disease (AOSD), as well as Systemic Onset Juvenile Idiopathic Arthritis (SOJIA, onset <16 years), which shows symptoms and biological mechanisms identical to the adult form.^95^ The ImmunAID consortium has already identified serum IL-18 as a biomarker that can discriminate Still’s disease from other SAID, while additionally found that levels of free IL-18 significantly decrease after treatment and may be relevant to assess response to therapy in these patients.^96^ Further, Dr. Soumelis’ team aimed to develop a transcriptome-based classification to capture disease heterogeneity. Instead of examining individual genes, his team analysed “features”, which are data-driven and knowledge-driven modules representing gene sets. Overall, 58 data-driven interpretable modules could capture differences between patients and controls. Additionally, 17 features of immune cell population signatures and 15 features of cytokine activation scores were used to identify five transcriptional subtypes of Still’s disease, each characterised by distinct immune patterns. This new molecular taxonomy highlights novel targets and provides a potential framework for personalised therapy and patient classification.

The second study examined idiopathic recurrent pericarditis (IRP), a rare condition characterised by recurrent inflammation of the pericardial layers.^97^ Dr. Soumelis nicely summarised the currently available therapeutic options for these patients. As a first-line treatment, IRP patients are treated with Nonsteroidal Anti-Inflammatory Drugs (NSAIDs), such as ibuprofen or aspirin, or colchicine that helps to reduce not only the symptoms but also the recurrence rates. As a second-line treatment, corticosteroids are administered in patients unresponsive to NSAIDs and colchicine. Finally, immunosuppressive therapy, such as azathioprine or canakinumab, and anakinra are given in refractory cases. Given the limited patient numbers suffering from IRP, Dr. Soumelis’ team adopted a sequential multi-omics approach combining plasma proteomics, bulk and single-cell transcriptomics, and flow cytometry. Plasma proteomics, performed by the Somascan platform, identified a peripheral inflammatory signature dominated by cytokine pathways. Bulk transcriptomics of PBMC identified 130 significantly regulated genes in IRP patients that were characterised by dominant inflammatory chemokine signatures. Flow cytometric analysis could pick up changes in immune cell populations in IRP patients. Finally, single cell RNA sequencing allowed the characterisation of cell-to-cell communications. Dr. Soumelis concluded that integrating multi-omics data enables mechanistic insights and the identification of potential biomarkers, ultimately advancing personalised treatment strategies for auto-inflammatory diseases.

Dr. Skendros explored how systemic non-infectious inflammatory diseases rarely fit neatly into either autoimmunity or autoinflammation, proposing instead a continuum in which the relative weight of adaptive versus innate immune activation can vary across diseases and even across stages of the same disease.^98^ He contrasted hallmark features of classical autoimmunity, such as loss of tolerance with autoreactive B and T cells and autoantibodies, with classical autoinflammation driven by innate immune cells and cytokine pathway deregulation, but emphasised that multiple layers of innate–adaptive cross-talk generate significant overlap in clinical phenotypes and molecular trajectories. In this context, he highlighted the practical challenge posed by fever and inflammation of unknown origin, in which infection, autoimmune rheumatic disease, and autoinflammatory disease may be complicated to distinguish early in the workup.^99^ As an example of pathway-guided diagnostics, he presented an interleukin-1β-DNA complex assay linked to neutrophil extracellular traps that distinguishes autoinflammatory disorders from autoimmune and infectious diseases with high accuracy and a clinically usable cutoff.^100^ Therapeutically, he connected this endotype-based thinking to targeted interventions, ranging from interleukin-1 pathway blockade and newer approaches to systemic autoinflammation to emerging B-cell-directed strategies such as CD19 CAR T-cell therapy in severe systemic autoimmune disease.^12,101^ He concluded with a unifying message: systemic non-infectious inflammatory diseases constitute a single pathophysiological landscape rather than two distinct categories, and moving toward a molecular systems-based classification beyond the conventional autoimmune–autoinflammatory dichotomy may enable more precise diagnosis and genuinely tailored therapies aligned with the dominant pathway in each patient.^102^

### Immune regulatory networks in autoimmunity and cancer

Prof. Mauri’s presentation explored the dual role of B cells in cancer immunity, highlighting the discovery of an immunoregulatory subset of double-negative (DN)1 B cells that suppress antitumor cytotoxic responses in renal cell carcinoma (RCC). B cells are known to exert both effector and regulatory functions. More specifically, while effector B cells promote inflammation through proinflammatory cytokines, regulatory B cells (Bregs) counterbalance immune activation by releasing IL-10, TGF-β and IL-35.^103–105^ This immunosuppressive capacity, although protective in autoimmunity, can support tumour progression by dampening effective T cell responses.

Using an integrative single-cell transcriptomic and computational approach, Prof. Mauri’s team addressed the limitations of previous pan-cancer B cell atlases, which merged data from multiple cancer types and masked tissue-specific differences.^106,107^ They, therefore, developed a novel bioinformatic pipeline to analyse tumour-infiltrating B cells within each cancer type independently, before integrating them into a shared space. This analysis revealed distinct, cancer-specific B cell clusters, including a prominent enrichment of DN1 B cells in RCC.

Validation using scRNA-seq and flow cytometry of RCC samples confirmed DN1 B cells as CD27^−^IgD^−^ cells localised within the tumour and peritumoral regions. Spatial imaging demonstrated that DN cells reside predominantly within immature tertiary lymphoid structures (TLSs) that lack germinal centres, characterised by disorganised follicular dendritic cells and sparse T follicular helper cells. Clinically, DN1 B cell enrichment correlated with poor prognosis, in contrast to DN3 and plasma cell signatures, which associated with improved outcomes. Of interest, DN (CD27^−^IgD^−^) B cells have been previously associated to the development of several autoimmune diseases.^108,109^

Further mechanistic analyses revealed that DN1 Bregs express a regulatory gene signature and interact closely with CD8^+^ T cells, inducing their functional suppression through IL-10 and TGF-β signalling. Gene set enrichment analyses implicated TLR7/9 activation as a driver of DN1 expansion, likely in response to damage associated molecular patterns from necrotic tumour cells. This work provides novel insights into B cell-mediated immune regulation in RCC.

Dr. Alissafi focused on immune regulatory networks and how their metabolic control could be harnessed to restore self-tolerance in autoimmune diseases. Initially, she highlighted the delicate balance between immune activation and regulation, and how excessive activation drives autoimmunity, whereas excessive regulation favours tumour development. Regulatory T cells are central to this balance, acting as the main guardians of self-tolerance, however, alterations in Treg number or function can impair their suppressive capacity and drive disease.^110^

In previous work, Dr. Alissafi has emphasised how metabolism is critical for Treg suppressive function. Under steady-state conditions, Tregs rely on mitochondrial metabolism and oxidative phosphorylation (OXPHOS) to maintain regulatory activity. In autoimmune settings, however, impaired mitochondrial function and mitochondrial oxidative damage underlie regulatory T cell defects.^111^

The focus then shifted to dendritic cells, the key orchestrators of immune responses. Their functional versatility stems from their heterogeneity, including subsets such as plasmacytoid DCs and conventional DCs, as well as differences between resident and migratory populations. Under homeostatic conditions, DCs sustain immune tolerance, but their dysregulation can trigger autoimmune pathology.^112^ However, despite their importance their heterogeneity and its functional importance for the pathogenesis of autoimmune diseases remain largely undefined. To address this gap, Dr. Alissafi’s group applied an integrative single cell multi-omic approach. Their findings identify novel dendritic cell checkpoints that can be evaluated for therapeutic targeting.

Dr. Alexopoulos presented an overview of Paraneoplastic Neurological Syndromes (PNS), focusing on their etiopathogenesis and the role of the tumour microenvironment (TME) in their development. PNS are immune-mediated neurological syndromes that occur in association with cancer, during which the anti-tumour immune response inadvertently targets neuronal antigens.^113^ The occurrence of these syndromes, being the result of a complex interplay between the immune system, cancer, and the nervous system, raise key questions regarding why only specific tumours trigger them and why the autoimmune response selectively affects the nervous system.

Dr. Alexopoulos first distinguished two major pathogenetic groups of PNS. The first involves antibodies targeting intracellular/onco-neuronal antigens, in which immune-mediated neuronal injury is primarily T cell dependent. These disorders are strongly cancer-associated and generally respond poorly to immunotherapy. The second group involves antibodies that target cell surface/synaptic antigens that are directly pathogenic, causing receptor internalisation or blockade. These forms often respond better to tumour removal and immunotherapy.^114^

The auto-immunisation process remains incompletely understood but likely involves B cell tolerance defects combined with a trigger factor such as tumours, viral infections or neurodegeneration. In brief, antigens released by tumour cells can reach the lymph nodes, where they activate autoreactive T and B cells, leading to antibody production.^115^ Notably, autoantibodies may persist for years even in the absence of symptomatology, implying that additional factors like blood-brain barrier breach might be needed for pathology.^116^ Moreover, no difference in epitope specificity was observed among the spectrum of anti- glutamic acid decarboxylase (GAD)-associated syndromes despite distinct clinical manifestations, suggesting that the auto-antibodies may not be pathogenic in autoimmune neurological diseases.^117^ Particular emphasis was given by the speaker to anti-Yo and anti-Ri syndromes. These two syndromes, both associated with breast cancer, exemplify the diversity of PNS. Despite their shared oncological background, they involve distinct neuronal antigens, and it remains unclear why patients develop different immune responses and consequently, distinct neurological manifestations.

In the final part of his talk, Dr. Alexopoulos presented novel data on the role of cancer-associated fibroblasts (CAFs) as potential contributors to auto-immunisation. CAFs are cells within the tumour microenvironment involved in tumour growth, angiogenesis, metastasis and immunomodulation.^118^ A distinct subset, termed antigen-presenting CAFs (apCAFs) can express MHC class II molecules and contribute to the organisation of tertiary lymphoid structures, thereby promoting local T cell and B cell activation.^119,120^ Preliminary findings suggest that apCAFs may also express onconeural antigens, raising the possibility that apCAF and TLS signatures could serve as biomarkers of PNS risk and potential targets for therapeutic intervention.

### Neutrophils as Drivers and Modulators of Disease

Dr. Galani’s presentation focused on the role of neutrophils in health and disease. Neutrophils are the most abundant leukocytes in circulation, short-lived and rapidly recruited at sites of inflammation. While their importance in bacterial and fungal infection is well-established, their role in viral infections remains poorly defined. Using an in vivo mouse model of Influenza A Virus (IAV) infection, Dr. Galani demonstrated that neutrophil infiltration in bronchoalveolar lavage (BAL) fluid and lung tissue increases as infection progresses, correlating with disease severity.^121^ Similarly, high neutrophil numbers and an elevated neutrophil-to-lymphocyte ratio has been observed in critically ill patients with IAV and COVID-19.^122^

Neutrophil depletion experiments revealed that although complete depletion of neutrophils in an IAV infection model worsens the outcome, their partial depletion improves survival.^123^ These results underscore that neutrophils are vital for viral control but can be harmful when excessively activated, pinpointing the importance of a balanced neutrophilic response during the anti-viral response. This is central to the most severe complication of a severe infection, the acute respiratory distress syndrome (ARDS), where numerous therapeutic strategies targeting individual neutrophil mechanisms have failed, likely due to the multifaceted nature of the neutrophilic response.^124,125^

To address this, Dr. Galani proposed that therapies should instead modulate upstream regulators of neutrophil activation, and presented data on two such regulators: Interferons and Activin A. Of particular interest, she discovered that neutrophils highly express type III interferon receptor (IFNLR), signalling via which initiates a distinct transcriptional program in neutrophils than the one triggered by type I IFNs, with the first one selectively promoting antiviral genes, while the second one triggering both antiviral and proinflammatory responses. Collectively, the data show that when there is a low viral load, IFN-λ is important to combat initially the infection without promoting inflammation but, if there is a severe infection, then type I IFNs are needed for antiviral defence, however at the same time they cause host damage. Importantly, administration of IFN-λ in infected mice improved survival, reduced viral load and decreased lung inflammation.^121^ Consistent with these findings, clinical studies during COVID-19 showed that pegylated IFN-λ treatment reduced hospitalisation time by half, underscoring its therapeutic potential.^126^

A second regulatory pathway involves Activin A, a member of the TGF-β superfamily. Knockout of Activin-A in neutrophils of transgenic mice led to excessive NET formation and worsened survival in infected animals, suggesting that Activin A acts as a brake on neutrophil-mediated pathology.^127^

Finally, Dr. Galani presented emerging data on chronic respiratory diseases, identifying pathogenic neutrophil subsets in bronchiectasis that correlate with disease severity. Moreover, she showed transcriptomic similarities between neutrophils from idiopathic pulmonary fibrosis and COVID-19, suggesting that there is a proinflammatory signature induced in lung neutrophils that is independent of the type of the disease.^128^

Dr. Kambas’ talk (“NETs in Inflammation”) provided a concise, mechanism-to-disease overview of NETs, from their discovery to their expanding role as potential “common pathogenic mechanisms” across inflammatory settings, thromboinflammation, autoimmunity, autoinflammation, fibrosis, and cancer. He outlined primary triggers, including phagocytosis, chemokine and complement signals, platelet-driven pathways, and ANCA, as well as key regulatory axes involving reactive oxygen species, myeloperoxidase and neutrophil elastase activity, autophagy, PAD4-mediated histone citrullination, and NET clearance efficiency.^129,130^ He then connected these pathways to rheumatology, emphasising that NETs can provide immunogenic material and amplify inflammation in SLE through plasmacytoid dendritic cell activation and type I interferon induction, while impaired NET degradation correlates with more severe organ involvement.^131,132^ In rheumatoid arthritis, NETs have been highlighted as a source of citrullinated autoantigens. As drivers of inflammatory amplification, NETs have been demonstrated in Giant Cell Arteritis (GCA) biopsies in association with inflammatory cytokine milieus.^133,134^ Overall, he emphasised that NET heterogeneity is essential for both biomarker interpretation and future NET-targeted therapeutic strategies.

### Work in progress by young investigators

Dr. Papadaki presented new work of Dr. Andreakos’ lab on lipid mediators (LMs) and their potential role as key regulators of the inflammatory response and its resolution.^97^ LMs are rapidly produced bioactive lipids in response to infection or inflammatory insults, and can exert pro-inflammatory and/or pro-resolving actions.^135,136^ Dr. Papadaki showed that LM networks are profoundly altered in COVID-19, and can stratify patients according to disease severity.^137^ Among 55 LMs analysed, 20-HETE emerged as a promising prognostic biomarker for ICU admission and a potential therapeutic target for severe COVID-19 disease.^137^ Using lipidomics analysis, she further aims to characterise specialised pro-resolving mediator (SPM) networks in SAID and to define their diagnostic and prognostic potential. To this end, in the context of the ImmunnAID consortium ω3 and ω6-derived SPMs have been quantified in plasma and urine of SAID patients, in the exacerbation and the remission phase. These data are analysed in combination with transcriptomic data from cells isolated from SAID patients, as well as functional in vitro data, in order to identify abnormalities in SPM biosynthesis and to explore pharmacological strategies that promote resolution of inflammation in SAIDs. This work, together with Prof. Soumelis’ approach within ImmunAID, aims to uncover molecular mechanisms that control inflammation and identify new therapeutic pathways for autoinflammatory diseases.

Dr. Palamidas investigated immune and LM dynamics in GCA and Polymyalgia Rheumatica (PMR), two closely related autoimmune/autoinflammatory disorders that respond rapidly to glucocorticoid treatment.^138^ His study aimed to identify novel next-generation biomarkers that reflect disease activity, organ damage and therapeutic response. Using multiparametric approaches, including high-dimensional immune profiling by CyTOF, multiplex cytokine analysis and targeted lipidomics, the team analysed sequential blood samples from GCA and PMR patients during diagnosis and early stages of steroid treatment (48, 96h), as well as a later time-point at 24 weeks.^139^ This design allowed the detection of rapid immunological changes associated with clinical improvement, as well as shifts in the inflammatory milieu. More specifically, changes in CD4+ T cells, CD8+ T cells and DCs, and subpopulations/ sub-phenotypes of these major immune cell types were highlighted (139). Regarding, cytokine analysis, GCA and PMR patients exhibited a similar cytokine profile during diagnosis, and it appeared that in the early phases of glucocorticoid treatment, cytokines and chemokines were not sensitive-to-change molecules, with the exception of ITAC, suggesting that the CXCR3-CXCL11 axis may play a role in the rapid clinical improvement of these patients.^139^ Of interest, lipidomics analysis showed that the ratio of pro/anti-inflammatory LMs was high at 48h after glucocorticoid treatment in patients with either PMR or GCA; however, 6 months after steroid treatment it returned to normal in PMR patients, but remained high in GCA patients, providing the only discriminatory element between the two diseases. In conclusion, this study provided a foundation for understanding rapid steroid responses in GCA and PMR and for the identification of novel biomarkers for disease activity and treatment monitoring.^139^

Building on GCA, ongoing work from Mr. Spyropoulos investigates tissue-level mechanisms of vascular inflammation in GCA, and how these mechanisms are affected under Tocilizumab treatment. In particular, his study focused on the role of NETs and senescent cells in GCA, two components that are linked with acute and chronic inflammation respectively. To this purpose, sequential temporal artery biopsies were analysed using immunofluorescence staining, confocal microscopy and advanced image analysis to quantify immune cell populations, NET formation and senescent cell presence. The rationale is to understand which inflammatory components respond rapidly to IL-6 receptor blockade and which resist in the vessel wall, providing insight into the paradox of clinical remission despite residual tissue inflammation. Mr. Spyropoulos concluded that NET presence in GCA biopsies correlated strongly with systemic markers and histologic activity, making them promising biomarkers of ongoing inflammation in GCA.

Dr. Panagopoulos’ presentation focused on pulmonary involvement in SjD, a condition traditionally defined by periductal lymphocytic infiltration of epithelial tissues and B cell hyperactivity leading to autoantibody production. Beyond the classic glandular manifestations, pulmonary manifestations represent a significant systemic component of the disease. These may include upper respiratory tract involvement manifested as xerotrachea, small airways disease (SAD), cystic lung disease and interstitial lung disease (ILD).^140,141^ Dr. Panagopoulos therefore aimed to describe the clinical landscape of pulmonary disease in SjD, to estimate the prevalence of SAD in SjD, and to investigate its potential relationship with ILD. To this end, he analysed 83 SjD patients with respiratory symptoms and/or asymptomatic SjD patients with abnormal pulmonary functional tests, and assessed pulmonary and small airways function, via classic spirometry, measurement of diffusing capacity for carbon monoxide (DLCO) and impulse oscillometry, combined with high-resolution computed tomography imaging of the lungs and questionnaires for respiratory symptoms.^142^ He reported a significant prevalence of ILD and SAD findings on HRCT, as well as small airways dysfunction by spirometry and impulse oscillometry. Of importance, SjD patients with ILD manifestations presented significantly higher minor salivary gland biopsy focus score than patients without ILD, suggesting a potential pathophysiologic link.

Following the discussion on pulmonary manifestations, Dr. Koutsogianni presented another facet of systemic involvement in SjD, the Raynaud’s phenomenon (RP).^143^ Affecting up to one-third of patients,^144^ RP may reflect microvascular dysfunction linking SjD to the scleroderma spectrum. Dr. Koutsogianni aimed to define distinct clinical and serological phenotypes of RP in SjD, focusing on the presence of anticentromere antibodies (ACA). Using data from a large multicentre cohort, comprising 1583 patients, individuals with and without RP were compared, and among those with RP, ACA-positive and ACA-negative groups were further analysed. Clinical, immunological and nailfold capillaroscopic features were assessed to explore associations with disease activity, with the results defining different phenotypes of RP in SjD. Dr. Koutsogianni reported that compared to SjD/RP- patients, SjD/RP+ patients had a higher prevalence of several other clinical manifestations, such as lymphoma and arthralgias, while among the RP+ patients, ACA-positivity characterises a more aggressive clinical course resembling limited scleroderma. Therefore, recognising subgroups within SjD is clinically meaningful, as it may have important implications for early diagnosis, risk stratification, and personalised follow-up.

Further exploring the diagnostic challenges in SjD, Dr. Panagiotopoulos presented his work employing deep learning for the automated evaluation of minor labial salivary gland biopsies.^145,146^ Given the variability and subjectivity in calculating the Focus Score, a key histopathologic criterion in SjD diagnosis, the study aimed to develop a machine learning model capable of objective and reproducible assessment. Using a weakly supervised deep learning approach trained on 271 digitised hematoxylin and eosin-stained biopsy slides, the model integrated preprocessing, feature extraction and attention-based classification to distinguish biopsies and calculate the FS. This proof-of-concept study demonstrates the potential of AI-driven histopathology to minimise interobserver variability, support diagnostic accuracy and facilitate consistent evaluation across centres. Importantly, such an automated approach could also serve as a valuable tool for underserved areas, helping to guide evaluation.

To conclude, Mr. Piperakis of Dr Andreakos’ lab described a newly generated *IFNLR1^tdTomato-cre^* reporter mouse model, which is designed to map IFNLR1 expression in vivo via the expression of the tdTomato fluorochrome. While type III interferons (IFNs) primarily act on epithelial cells at barrier surfaces to mediate anti-viral protection,^147,148^ IFNLR1 expression has also been reported in circulating and splenic neutrophils.^121,149^ Validation of the mouse model through histological analysis confirmed tdTomato fluorescence in lung and intestinal epithelial cells, while flow cytometric analysis demonstrated high tdTomato expression in splenic and blood neutrophils. These findings are consistent with previous studies and indicate that this reporter mouse is a useful and reliable tool for studying IFNLR1 expression across tissues and immune cell populations.

## DISCUSSION

Overall, the second meeting reaffirmed the value of sustained dialogue between clinicians and basic scientists in an era of rapidly evolving, high-dimensional biomedical technologies. By centring discussions on unmet clinical needs and grounding mechanistic insights in real-world patient care, the symposium helped reanimate the clinician–scientist paradigm and narrow the translational divide. The strong engagement of participants and the depth of interdisciplinary exchange underscored the meeting’s role as a catalyst for meaningful collaboration, positioning it as a cornerstone initiative for driving innovation, shaping future research directions, and ultimately improving outcomes for patients with systemic autoimmune diseases.
